# A ten-year case series of rare specific phobias in an outpatient psychiatric clinic at a regional hospital

**DOI:** 10.1192/j.eurpsy.2026.11222

**Published:** 2026-07-31

**Authors:** F. Zaouali, I. Anes

**Affiliations:** 1 Emergency department and mobile emergency and resuscitation service, Tahar Sfar University Hospital, Mahdia; 2 Outpatient psychiatric consultation, Hadj Ali Soua Regional Hospital, Ksar Hellal, Monastir, Tunisia

## Abstract

**Introduction:**

Specific phobias, characterized by excessive and persistent fear of particular objects or situations, are common anxiety disorders. However, rare forms of specific phobias remain underreported in clinical practice and literature. Understanding these uncommon presentations is crucial for timely diagnosis and effective management.

**Objectives:**

This study aims to describe the clinical characteristics, therapeutic approaches, and outcomes of patients presenting with rare specific phobias over a ten-year period in a regional psychiatric outpatient setting (2013-2023).

**Methods:**

A retrospective review was conducted of medical records from the psychiatric outpatient consultation at hadj Ali Soua regional hospital, identifying cases diagnosed with rare specific phobias from January 2013 to December 2023. Inclusion criteria encompassed phobias with low prevalence such as phobophobia, trypanophobia, or phobias related to unusual stimuli. Data collected included demographic information, phobia subtype, comorbid psychiatric disorders, treatment received (psychotherapy, pharmacotherapy), and clinical follow-up outcomes.

**Results:**

Twelve cases meeting inclusion criteria were identified, with patients aged 16 to 52 years (mean age 33.1 ± 9.4), including 7 females and 5 males. Phobia subtypes included fear of needles (trypanophobia, 5 cases), fear of choking (2 cases), fear of vomiting (emetophobia, 2 cases), fear of mirrors (spectrophobia, 1 case), and phobophobia (fear of phobias, 2 cases). Comorbidities were common, including generalized anxiety disorder and depressive disorders. Cognitive-behavioral therapy (CBT) was the mainstay treatment in 10 cases, sometimes combined with selective serotonin reuptake inhibitors (SSRIs). Follow-up over an average of 18 months showed partial to full remission in 8 cases, while 4 cases reported persistent symptoms requiring ongoing care. The findings underscore the heterogeneity and clinical complexity of rare phobias.

**Conclusions:**

Rare specific phobias, though infrequent, pose significant challenges in diagnosis and management within outpatient psychiatric services. A tailored approach combining psychotherapy and pharmacotherapy yields favorable outcomes for most patients. Increased awareness and systematic documentation are essential for advancing understanding and treatment of these conditions. Further research with larger cohorts is warranted.

**Disclosure of Interest:**

None Declared

